# Severe fever with thrombocytopenia syndrome complicated by invasive pulmonary aspergillosis and septic shock: a case report highlighting the role of mNGS

**DOI:** 10.3389/fmed.2026.1888410

**Published:** 2026-07-22

**Authors:** Bolin Wang, Mingyue Zhao, Qingfeng Chen, Feihu Zhang, Mingzhi Fan, Xinbao Lian

**Affiliations:** 1 Affiliated Hospital of Shandong University of Traditional Chinese Medicine (First Clinical Medical College of Shandong University of Traditional Chinese Medicine), Jinan, Shandong, China; 2 Jinan Seventh People's Hospital, Jinan City, Shandong Province, China; 3 Shandong Provincial Police General Hospital, Jinan City, Shandong Province, China

**Keywords:** case report, intensive care, invasive pulmonary aspergillosis, metagenomic next-generation sequencing, severe fever with thrombocytopenia syndrome

## Abstract

**Background:**

Severe fever with thrombocytopenia syndrome (SFTS) is an emerging tick-borne viral hemorrhagic fever associated with high mortality, and no specific antiviral therapy is currently available. Patients with SFTS often develop immune dysfunction, rendering them susceptible to secondary opportunistic infections, particularly invasive pulmonary aspergillosis (IPA). Early diagnosis of this co-infection is critical but remains challenging due to nonspecific clinical manifestations and radiological findings.

**Case presentation:**

A 61-year-old male farmer from a hilly region presented in July 2024 with fever, dyspnea, and altered consciousness. On admission, he exhibited septic shock and multiple-organ dysfunction, including severe thrombocytopenia, leukopenia, liver injury, and acute kidney injury. Metagenomic next-generation sequencing (mNGS) of blood and bronchoalveolar lavage fluid rapidly identified SFTS virus, *Aspergillus fumigatus*, Aspergillus flavus, and multiple Gram-negative bacteria. Chest imaging revealed bilateral nodules distributed along the bronchovascular bundles, suggestive of angioinvasive IPA. Treatment consisted of imipenem/cilastatin, isavuconazonium sulfate, continuous renal replacement therapy, and mechanical ventilation. The patient gradually improved and was discharged after 30 days, with complete clinical recovery documented at the 3-month and 9-month follow-up visits.

**Conclusion:**

This case highlights the diagnostic value of mNGS in critically ill patients with SFTS and suspected co-infections, as it enables early pathogen identification and targeted therapy. Clinicians in endemic areas should maintain a high index of suspicion for SFTS and IPA in patients presenting with unexplained fever, thrombocytopenia, and organ dysfunction. However, the favorable outcome cannot be attributed solely to mNGS, as multiple supportive interventions were administered concurrently; the clinical improvement likely reflects a synergistic effect of timely targeted therapy and comprehensive intensive care.

## 1 Introduction

Severe fever with thrombocytopenia syndrome (SFTS) is a viral hemorrhagic fever caused by *Dabie bandavirus* (DBV) (1) and is primarily transmitted through bites from *Haemaphysalis longicornis* ticks. In recent years, the endemic range of SFTS has progressively expanded across East Asia. SFTS is clinically characterized by fever, thrombocytopenia, leukopenia, gastrointestinal symptoms, and multiple-organ dysfunction. The management of SFTS remains challenging due to the absence of specific vaccines, the lack of effective antiviral therapies, and the high case fatality rate. Evidence indicates that DBV compromises host antiviral immunity by disrupting both innate and adaptive immune responses and altering immune cell numbers and function (2). Furthermore, DBV can affect multiple cellular signaling pathways by inhibiting type I interferon (IFN) signaling, modulating nuclear factor kappa-light-chain-enhancer of activated B cells (NF-κB) signaling, and interfering with autophagy. These mechanisms further weaken host antiviral immune responses, thereby facilitating viral replication and dissemination (3).

Due to DBV-induced immune dysregulation, patients with SFTS are highly susceptible to secondary opportunistic infections, particularly invasive pulmonary aspergillosis (IPA), which has been associated with mortality rates of up to 22.0% (4). Moreover, this immune dysregulation may trigger a cytokine storm, leading to systemic inflammatory response syndrome (SIRS) and multiorgan damage (5).

In North America, human granulocytic anaplasmosis (HGA), caused by *Anaplasma phagocytophilum*, may resemble SFTS clinically, presenting with fever, pancytopenia, elevated liver enzyme levels, and multiorgan failure (6–8). A recent systematic review reported thrombocytopenia in 71.8% of anaplasmosis cases, elevated liver enzyme levels in 66.7%, and leukopenia in 49.8%, with severe complications including acute renal failure (9.8%) and multiorgan failure (7.5%) (8). Therefore, clinicians should consider anaplasmosis in the differential diagnosis of patients presenting with febrile pancytopenia and multiorgan dysfunction, particularly those with a history of tick exposure in endemic regions (9). The clinical presentation of SFTS—fever, thrombocytopenia, leukopenia, and gastrointestinal symptoms—is nonspecific and can be mimicked by a wide range of infectious diseases, including hemorrhagic fevers (e.g., dengue, hantavirus infection), rickettsial diseases (e.g., scrub typhus, anaplasmosis), leptospirosis, severe bacterial sepsis, and hemophagocytic lymphohistiocytosis (HLH). These conditions may coexist with or complicate the course of SFTS, particularly in critically ill patients, making accurate and timely differential diagnosis a significant clinical challenge. Delayed recognition of the underlying etiology may lead to inappropriate empirical therapy, missed diagnosis of secondary opportunistic infections such as invasive pulmonary aspergillosis, and ultimately progression to multiorgan failure and death. Conversely, early identification of the specific pathogen(s)—whether viral, bacterial, or fungal—enables targeted antimicrobial or antifungal therapy, avoids unnecessary immunosuppression, and guides supportive care strategies. The use of advanced molecular tools, such as metagenomic next-generation sequencing, has markedly improved diagnostic accuracy and turnaround time in complex cases. Therefore, a high index of suspicion for alternative and concurrent diagnoses, combined with prompt and comprehensive microbiological investigation, is essential to improve clinical outcomes in patients presenting with suspected SFTS.

## 2 Case description

A 61-year-old man was admitted to the emergency department on July 13, 2024, with a 3-day history of fever and a 1-day history of dyspnea and altered consciousness. On presentation, his temperature was 38.9 °C and his heart rate was 120 beats per minute; his main symptoms included fever, chills, and tachypnea. He had initially been evaluated at Jining First People’s Hospital, where investigations led to a preliminary diagnosis of severe pneumonia, type 1 respiratory failure, and suspected myelodysplastic syndrome. He was subsequently referred to the emergency department of our institution. On admission, the patient exhibited shock and hypoxemia, necessitating mechanical ventilation and immediate transfer to the intensive care unit.

On admission, his vital signs were as follows: temperature, 38.1 °C; blood pressure, 144/79 mmHg; heart rate, 100 beats per minute; respiratory rate, 30 breaths per minute; and blood glucose level, 6.1 mmol/L. The patient was confused and agitated, presenting with high fever, diaphoresis, tachypnea, and apparent distress. Physical examination showed no skin rash, petechiae, or ecchymosis. The mucous membranes were moist, with no evidence of bleeding. No palpable cervical, axillary, or inguinal lymphadenopathy was detected. Chest auscultation revealed diminished breath sounds in both lower lung fields, with bilateral dry rales. Abdominal examination revealed a soft, non-tender abdomen, with no hepatosplenomegaly or guarding. Bowel sounds were normal. Neurological examination revealed no nuchal rigidity, Kernig’s sign, or focal neurological deficits; the patient remained confused and agitated, with a Glasgow Coma Scale (GCS) score of 14 (E4V4M6). Muscle strength was preserved in all four limbs, and deep tendon reflexes were symmetrical. The remainder of the physical examination was otherwise unremarkable. A detailed medical history revealed that the patient was a farmer who had worked in the fields before symptom onset. He reported having fallen in the rain and aspirated muddy water. He had no significant medical history or family history of genetic disorders and denied smoking or alcohol use.

Arterial blood gas analysis indicated metabolic acidosis, with the following results: pH, 7.30; PCO₂, 35.4 mmHg; PO₂, 59.1 mmHg; Na^+^, 137.5 mmol/L; K^+^, 3.45 mmol/L; Ca^2+^, 1.08 mmol/L; Cl^−^, 112.4 mmol/L; glucose, 4.0 mmol/L; bicarbonate, 18.2 mmol/L; total hemoglobin, 14.6 g/dL; lactate, 2.7 mmol/L; base excess, −6.81 mmol/L; and anion gap, 10.3 mmol/L. Complete blood count revealed the following: white blood cell count, 1.94 × 10^9^/L; absolute lymphocyte count, 0.8 × 10^9^/L; hemoglobin level, 142 g/L; platelet count, 30 × 10^9^/L; and neutrophils, 55.2%. Biochemical testing showed the following: blood urea nitrogen, 8.83 mmol/L; creatinine, 99 μmol/L; C-reactive protein, 37 mg/L; albumin, 26.6 g/L; and procalcitonin, 9.08 ng/mL. Liver function tests were notable for alanine aminotransferase, 114 U/L; aspartate aminotransferase, 434 U/L; total bilirubin, 21 μmol/L; and direct bilirubin, 11.4 μmol/L. Coagulation tests showed the following: international normalized ratio, 1.09; prothrombin time, 14.2 s; activated partial thromboplastin time, 75.2 s; plasma fibrinogen, 2.36 g/L; and plasma D-dimer, 3.62 μg/mL. Urinalysis was positive for ketones (+) and protein (+++). The serum galactomannan (GM) assay for Aspergillus yielded a value of 0.72 S/CO, and the GM assay of bronchoalveolar lavage fluid yielded 3.55 S/CO. Lymphocyte subset testing indicated immunosuppression. According to the 2024 FUNICU criteria (10), invasive aspergillosis was clinically suspected.

**Figure 1:**
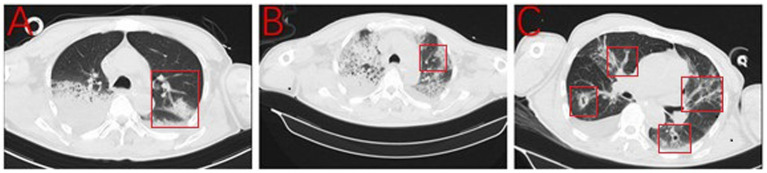
Serial chest CT findings during hospitalization. **(A)** Day 1: bilateral posterior high-density opacities and bilateral pleural effusion. A halo sign was observed. **(B)** Day 9: increased bilateral patchy high-density opacities with greater lesion extent and increased bilateral pleural effusion compared with the previous scan. Consolidation was observed. **(C)** Day 16: decreased lesion extent in both upper lobes and the right middle lobe, with new cavitary lesions in both lower lobes and decreased bilateral pleural effusion compared with the previous scan. An air crescent sign was observed.

Contrast-enhanced computed tomography (CT) of the chest, abdomen, and pelvis revealed bilateral posterior high-density pulmonary opacities and bilateral pleural effusion (Figure 1A). Importantly, no hepatic abscess or other focal liver lesions were observed, making hepatic aspergillosis unlikely despite the patient’s liver function impairment. Initial microbiological cultures of sputum, urine, and blood were negative. Metagenomic next-generation sequencing (mNGS) of blood identified severe fever with thrombocytopenia syndrome virus (SFTSV) and *Haemophilus influenzae*. Subsequent mNGS of bronchoalveolar lavage fluid (BALF) identified SFTSV, *H. influenzae*, *Aspergillus flavus*, *Aspergillus fumigatus*, *Stenotrophomonas maltophilia*, *Klebsiella pneumoniae*, and *Acinetobacter baumannii* (Table 1).

**Table 1:** Metagenomic next-generation sequencing results.

Sample	Pathogen		
	Name	Normalized reads	Estimated pathogen concentration
Blood	SFTSV	169,579	10^5^
	*Haemophilus influenzae*	67	10^2^
BALF	SFTSV	111,037	10^4^
	*Haemophilus influenzae*	540,722	10^5^
	*Stenotrophomonas maltophilia*	6,407	10^4^
	*Klebsiella pneumoniae*	6,407	10^4^
	*Acinetobacter baumannii*	1,443	<10^3^
	*Aspergillus flavus*	553	<10^3^
	*Aspergillus fumigatus*	298	<10^3^

SFTSV, severe fever with thrombocytopenia syndrome virus; BALF, bronchoalveolar lavage fluid.

Normalized reads: For each sample, 1 million reads were subsampled; higher normalized read counts reflect stronger pathogen detection signals.

Estimated pathogen concentration: Semiquantitative estimates based on read counts and internal control copy numbers, for clinical reference only. Not absolute quantification.

Supplementary information: Additional methodological details for mNGS are included in the supplementary information and are available from the corresponding author upon reasonable request.

The final diagnosis was severe fever with thrombocytopenia syndrome complicated by invasive pulmonary aspergillosis, septic shock, and sepsis-associated type 1 respiratory failure.

Initial antimicrobial therapy consisted of imipenem/cilastatin, administered at 1 g every 8 h as a prolonged 2-h infusion, because the patient presented with septic shock and an elevated procalcitonin level of 9.08 ng/mL, suggesting bacterial co-infection. Standard supportive care, including fluid resuscitation, albumin supplementation, and organ support, was also provided. On hospital day 2, mNGS of blood and bronchoalveolar lavage fluid confirmed the presence of SFTS virus, *Aspergillus flavus*, *A. fumigatus*, and multiple Gram-negative bacteria. Therefore, isavuconazonium sulfate was added for targeted antifungal coverage against Aspergillus species, with a loading dose of 200 mg every 8 h for the first 48 h followed by 200 mg once daily. Platelet transfusion was initiated because the platelet count had decreased to 30 × 10^9^/L, indicating a high risk of bleeding. Within the first 24 h, the patient’s acid–base status normalized; however, the platelet count continued to decline and renal function worsened. By hospital day 4, serum creatinine had increased to 205 μmol/L, and progressive oliguria had developed, necessitating continuous renal replacement therapy (CRRT) for acute kidney injury and fluid overload.

**Figure 2:**
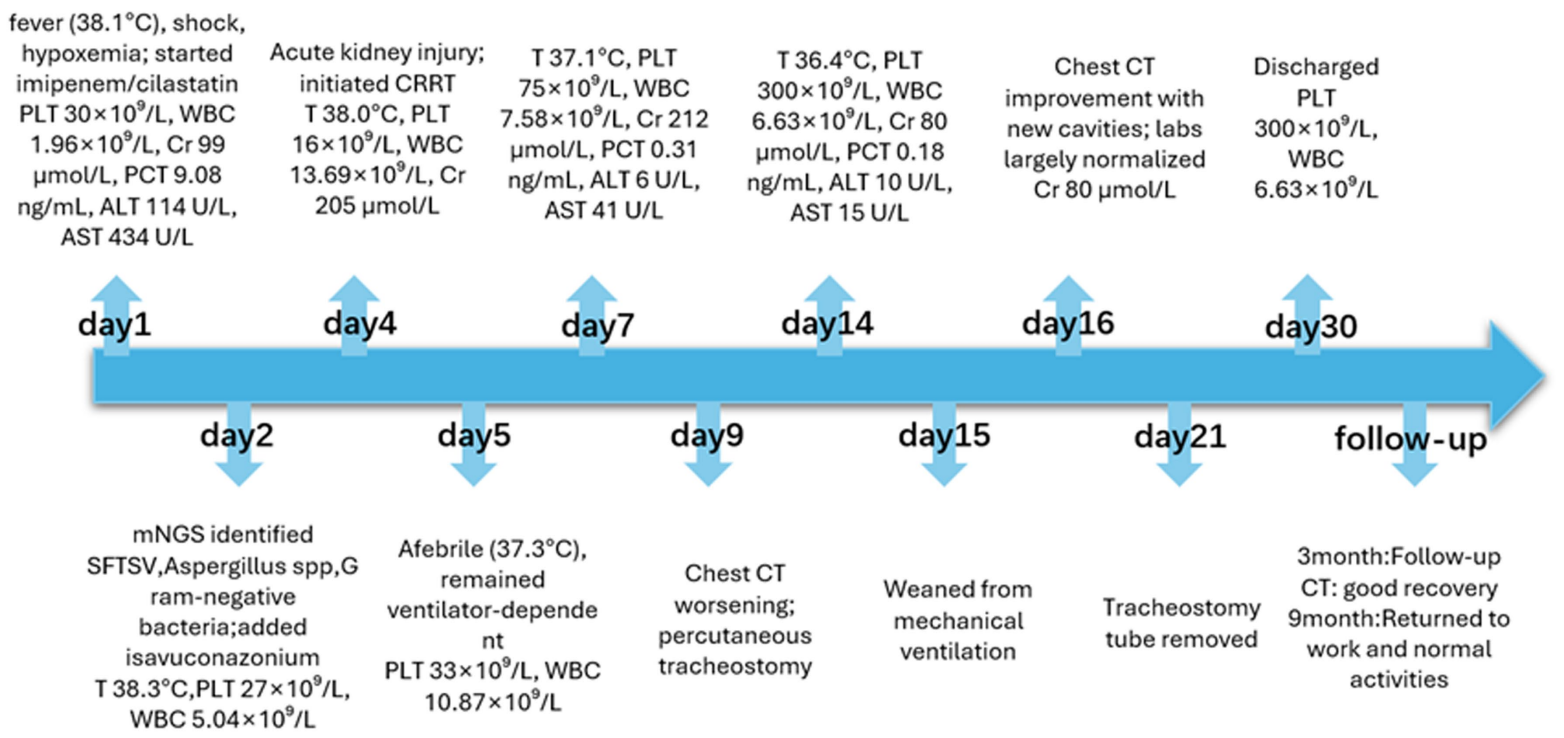
Timeline of clinical events and laboratory parameters. T, temperature; PLT, platelet count; WBC, white blood cell count; Cr, serum creatinine; PCT, procalcitonin; ALT, alanine aminotransferase; AST, aspartate aminotransferase; M, month.

After 5 days of antibiotic therapy, the patient became afebrile (temperature, 37.3 °C) but remained dependent on mechanical ventilation. Follow-up laboratory tests on day 5 showed improvements in white blood cell and platelet counts, which increased to 10.87 × 10^9^/L and 33 × 10^9^/L, respectively; however, chest CT on day 9 demonstrated worsening pneumonia and increased bilateral pleural effusions compared with previous imaging (Figure 1B). Because of suboptimal pulmonary recovery and persistently high ventilatory support requirements, percutaneous tracheostomy was performed after discussion with the patient’s family to facilitate ventilator weaning and airway management. The patient was successfully weaned from mechanical ventilation on hospital day 15. Chest CT on day 16 showed a reduced extent of pneumonia but new cavitary lesions in both lower lobes (Figure 1C). Laboratory parameters had largely normalized, and the patient was transferred to the general ward. The tracheostomy tube was removed, and the stoma was closed on hospital day 21; the patient was discharged home in good condition on hospital day 30. Follow-up laboratory results are shown in Figure 2.

The total treatment course included imipenem/cilastatin for 14 days, intravenous isavuconazonium sulfate for 12 days followed by a switch to oral therapy, and three therapeutic platelet transfusions.

## 3 Follow-up and outcome

**Figure 3:**
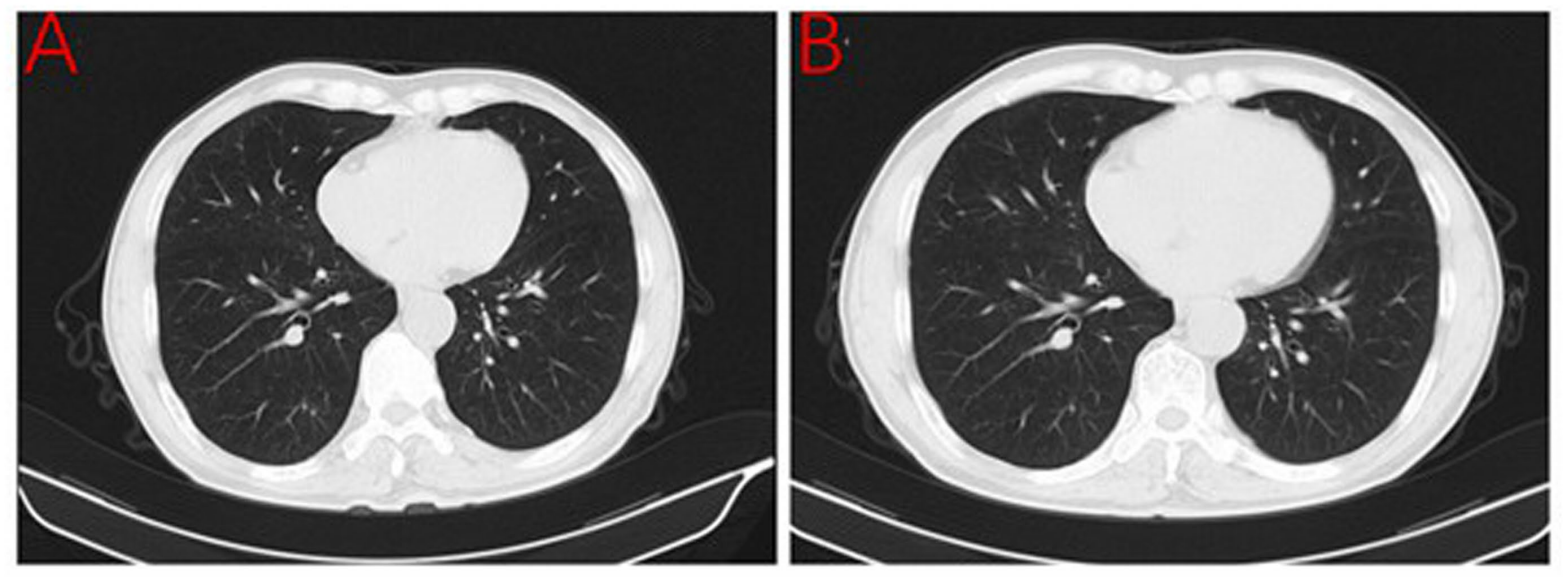
Follow-up chest CT findings. **(A)** At 3 months after discharge and **(B)** at 9 months after discharge: marked improvement compared with the initial admission CT findings.

At the 3-month and 9-month follow-up visits after discharge, chest CT showed near-complete recovery with no evidence of recurrence (Figure 3), and the patient had successfully returned to work and normal social activities. After discharge, the patient was interviewed and provided the following statement: “I was very weak and frightened when I first became ill. I had difficulty breathing and did not know what was happening. The doctors explained my condition to my family. After the tracheostomy and several weeks of treatment, I gradually recovered. I can now return to farming and live a normal life. I am grateful to the medical team for saving my life.” The patient also expressed satisfaction with the communication and care he received.

## 4 Discussion

Severe fever with thrombocytopenia syndrome (SFTS) is an emerging tick-borne viral disease associated with high mortality, and treatment options remain limited. Invasive pulmonary aspergillosis (IPA) is a notable complication in patients with SFTS-associated immune dysfunction and typically occurs during the progressive phase of SFTS, approximately 8–10 days after disease onset. In the present case, IPA was diagnosed early, on hospital day 2, using metagenomic next-generation sequencing (mNGS), which enabled prompt initiation of antifungal therapy.

First, classic radiographic features of IPA, such as cavitary lesions, the halo sign, and the air crescent sign, were absent. Instead, CT revealed multiple nodules distributed along the bronchovascular bundles, a pattern often described as angioinvasive IPA in immunocompromised hosts. However, in non-neutropenic patients, radiological-pathological correlations are often more heterogeneous, and mixed imaging patterns may coexist (7). Therefore, although the imaging findings in this case were consistent with angioinvasive IPA, definitive subclassification would require histopathological confirmation. Second, mNGS of blood and bronchoalveolar lavage fluid simultaneously identified SFTS virus, *Aspergillus fumigatus*, *A. flavus*, and multiple Gram-negative bacteria within 48 h of admission. This rapid and unbiased detection guided targeted antimicrobial therapy and helped avoid unnecessary antibiotic escalation (8). Third, despite impaired liver function, abdominal CT showed no evidence of hepatic aspergillosis, a finding consistent with the rarity of isolated hepatic aspergillosis in non-neutropenic patients.

A major challenge in managing this case was distinguishing true pathogens from colonizers or contaminants, as mNGS simultaneously detected multiple bacterial and fungal species with variable read counts. Current guidelines emphasize that mNGS results should never be interpreted in isolation but rather integrated with host factors, inflammatory markers, imaging, and treatment response (8). Given the polymicrobial nature of the findings, we adopted a hierarchical interpretative approach. High-read pathogens (*H. influenzae*, *K. pneumoniae*) with concordant clinical and laboratory signs (septic shock, PCT > 9 ng/mL) were considered true infections and guided the use of empirical broad-spectrum carbapenem therapy; this decision was further supported by the patient’s subsequent clinical improvement, although the possibility of overtreatment cannot be entirely excluded. Low-read organisms were evaluated on a case-by-case basis. For *Aspergillus* spp., although reads were low, the diagnosis of IPA was substantiated by a positive serum GM index (0.72), a markedly elevated BALF GM index (3.55), typical CT findings (nodules with subsequent cavitation), and a favorable clinical response to isavuconazonium. For *A. baumannii*, which had very low normalized read counts (<1 × 10^3^) and negative conventional cultures, no specific anti-Acinetobacter therapy was added, as imaging and the clinical course did not suggest invasive infection, and the findings were more consistent with colonization or contamination.

The patient’s history of muddy water aspiration and severe pneumonia prompted several differential considerations. Aspiration pneumonia was initially considered, but profound thrombocytopenia (30 × 10^9^/L) and leukopenia (1.94 × 10^9^/L) are atypical for uncomplicated aspiration pneumonia, and mNGS identified SFTSV with subsequent polymicrobial superinfection. Other viral, atypical bacterial, and *Pneumocystis* pneumonias were excluded by negative mNGS results and imaging correlation. Beyond pulmonary infections, fever with pancytopenia may also arise from systemic conditions. Human granulocytic anaplasmosis (HGA), which shares similar features (9), was excluded because it rarely causes marked leukopenia (<1.0 × 10^9^/L) or hemorrhage, and mNGS confirmed SFTSV as the primary pathogen. Hemophagocytic lymphohistiocytosis was ruled out by the absence of splenomegaly, hyperferritinemia, and hemophagocytosis (11). Other endemic tick-borne infections were excluded by the absence of eschar/rash and definitive SFTSV detection. Thus, mNGS-based comprehensive pathogen detection, combined with clinical and radiographic correlation, allowed systematic exclusion of alternative diagnoses and confirmed SFTS with IPA and bacterial co-infection as the definitive diagnosis.

Antiviral agents, including ribavirin and favipiravir, were not administered in this case. Although both agents have been investigated for the treatment of SFTS (12), several concerns precluded their use in this patient. First, the patient presented with significant liver injury (alanine aminotransferase level, 114 U/L; aspartate aminotransferase level, 434 U/L) and evolving acute kidney injury, with serum creatinine increasing from 99 μmol/L to 205 μmol/L by day 4. Ribavirin is associated with dose-dependent hemolytic anemia and requires renal dose adjustment, whereas favipiravir has been linked to hepatotoxicity and hyperuricemia. Given the lack of robust clinical trial evidence supporting antiviral therapy for SFTS and the potential risk of exacerbating organ dysfunction, a conservative therapeutic approach was adopted. Corticosteroids were also withheld because of positive mNGS results for Aspergillus and concerns regarding further immunosuppression (13). A review of the literature reveals that several other interventions have been explored for SFTS, including calcium channel blockers (14), intravenous immunoglobulin (15), plasma exchange (16), and monoclonal antibodies (17). However, most of the supporting evidence derives from *in vitro* studies or small case series. Therefore, the potential benefits of these interventions require further validation in well-designed clinical trials.

Of note, on March 25, 2026, the National Health Commission (NHC) of China issued an announcement stating that, effective April 1, 2026, severe fever with thrombocytopenia syndrome (SFTS) would be classified as a Class B notifiable infectious disease under the Law of the People’s Republic of China on the Prevention and Treatment of Infectious Diseases (18, 19). This regulatory upgrade highlights the growing public health importance of SFTS in China. SFTSV is primarily transmitted via tick bites, but mounting evidence indicates that human-to-human transmission can occur through direct contact with blood or bloody body fluids of infected patients, particularly in healthcare settings and family clusters (20–23). Importantly, no human-to-human transmission of SFTSV occurred during the treatment of this case, and no healthcare workers became secondarily infected.

This case underscores the need for clinicians in SFTS-endemic areas to maintain a high index of suspicion for IPA, even when typical CT signs are absent. In addition, early pathogen identification by mNGS enabled timely targeted antibacterial and antifungal therapy, which may have contributed to the patient’s clinical improvement.

## 5 Study limitations

This case report has several inherent limitations. First, because this is a single case report, the findings have limited generalizability and cannot establish causal relationships between specific interventions and clinical outcomes. The concurrent use of multiple therapeutic modalities—including broad-spectrum antibiotics, antifungal therapy, continuous renal replacement therapy, mechanical ventilation, and supportive care—makes it difficult to attribute clinical improvement to any single intervention, and particularly challenging to isolate the specific impact of mNGS-guided therapy on the final outcome. Second, although mNGS enabled rapid pathogen detection, the clinical significance of multiple organisms with low normalized read counts, including *A. baumannii* and Aspergillus species, remains uncertain; these findings may represent colonization or environmental contamination rather than true infection. Third, the absence of standardized diagnostic criteria for IPA specifically in patients with SFTS limits the diagnostic certainty of this co-infection. Collectively, these limitations suggest that our observations should be considered hypothesis-generating rather than confirmatory, and underscore the need for larger prospective studies to validate these findings.

## 6 Conclusion

We report a case of severe fever with thrombocytopenia syndrome complicated by invasive pulmonary aspergillosis, leading to sepsis and multiorgan dysfunction. In SFTS-endemic areas, particularly hilly and forested regions, SFTS should be considered in individuals presenting with unexplained fever, diarrhea, thrombocytopenia, and markedly elevated cardiac enzyme levels, regardless of reported tick bite history. Metagenomic next-generation sequencing (mNGS) may facilitate early and accurate diagnosis, thereby enabling timely treatment and potentially reducing mortality. Heightened clinical awareness and early diagnosis are crucial for improving outcomes in this insidious and life-threatening syndrome. This case highlights the diagnostic and therapeutic challenges of SFTS complicated by co-infection with other pathogens and emphasizes the importance of early clinical suspicion, comprehensive microbiological evaluation, and adaptive treatment strategies.

## Data Availability

The original contributions presented in the study are included in the article/Supplementary material, further inquiries can be directed to the corresponding author.
