# Supplementary material for: Severe fever with thrombocytopenia syndrome complicated by invasive pulmonary aspergillosis and septic shock: a case report highlighting the role of mNGS
Source: Front Med (Lausanne). 2026 Jul 22;13:1888410. doi: 10.3389/fmed.2026.1888410 (PMC13439413; doi:10.3389/fmed.2026.1888410)
Supplement: Supplementary file 1 [file Data_Sheet_1.PDF]

1. Nucleic Acid Extraction

DNA and RNA were extracted from each sample using the PathoXtract® Universal Nucleic Acid Extraction Kit (Magnetic Bead Method) (Cat. No. WYXM10002D-50, Weiyan Medical).

2. Library Preparation

RNA was reverse-transcribed into cDNA using the PathoXtract® RNA Purification Kit (Cat. No. WYCM06601D-24, Weiyan Medical). Subsequently, equal amounts of DNA and cDNA were pooled, and library construction was performed using the PathoLib™ Genomic Fragmentation Kit (Cat. No. WYLM044032D-96, Weiyan Medical) and the PathoLib™ Genomic Library Enrichment Kit (Cat. No. WYLM04407S-96, Weiyan Medical).

3. High-Throughput Sequencing

Sequencing was carried out on the MGISEQ-200 platform in single-end 50-bp mode, generating an average of 10 million reads per sample.

4. Data Quality Control

Raw FASTQ data were subjected to quality control and assessment using Trimmomatic v0.40. Low-quality reads, reads with undetermined bases, adapter-contaminated reads, high-duplication reads, and excessively short reads were filtered out to retain high-quality sequencing data. The high-quality reads were then aligned to the human reference genome GRCh37 (hg19) using Bowtie2 v2.4.3 to remove human-derived sequences, yielding clean data for subsequent pathogen identification. The clean data were aligned against a comprehensive pathogen database (NCBI GenBank/RefSeq/nt) using Kraken2 v2.1.0 for taxonomic annotation, thereby providing the final microbial identification results.

5. Positive Result Interpretation

The abundance of each microorganism was quantified as RPTM (reads per ten million – i.e., the number of microbial-specific reads detected per 10 million total sequencing reads). The positivity thresholds were set as follows: RPTM ⩾ 3 for viruses; RPTM ⩾ 20 for bacteria and fungi. For specific pathogens (i.e., *Cryptococcus* and *Mycobacterium tuberculosis*), an RPTM ⩾ 1 was reported as positive.

6. Overall Quality Control Measures

① Background microorganism information was monitored in real time. By integrating the detection results of negative controls from each batch over the preceding 30 days, a dynamically updated background database was constructed based on continuously accumulated sample data.

② Positive control detection was monitored in real time. The results of positive controls

in each batch were reviewed to identify any risks of false-negative or false-positive results arising from operational errors.
③ The detection of a positive internal control (spiked into each sample) was monitored in real time to assess the stability of the experimental procedure and to avoid false-negative outcomes.
④ Bioinformatics reference standards were established and used to iteratively optimize the pathogen database, thereby continually improving the performance of pathogen identification.

7. Turnaround Time

The sample turnaround time was 18–24 hours.
